# The Role of Autophagy in Chemical Proteasome Inhibition Model of Retinal Degeneration

**DOI:** 10.3390/ijms22147271

**Published:** 2021-07-06

**Authors:** Merry Gunawan, Choonbing Low, Kurt Neo, Siawey Yeo, Candice Ho, Veluchamy A. Barathi, Anita Sookyee Chan, Najam A. Sharif, Masaaki Kageyama

**Affiliations:** 1 Santen-SERI Open Innovation Centre, 20 College Road, The Academia, Singapore 169856, Singapore; merry.gunawan@santen.com (M.G.); choon.bing.low@santen.com (C.L.); kurt.neo@santen.com (K.N.); 2 Translational Pre-Clinical Model Platform, Singapore Eye Research Institute, 20 College Road, The Academia, Singapore 169856, Singapore; yeo.sia.wey@seri.com.sg (S.Y.); amutha.b.veluchamy@seri.com.sg (V.A.B.); 3 Singapore Eye Research Institute, 20 College Road, The Academia, Singapore 169856, Singapore; candice.ho.e.h@seri.com.sg (C.H.); anita.chan.s.y@singhealth.com.sg (A.S.C.); 4 Department of Ophthalmology, Yong Loo Lin School of Medicine, National University of Singapore, 21 Lower Kent Ridge Road, Singapore 119077, Singapore; 5 Academic Clinical Program in Ophthalmology, Duke-NUS Medical School, 8 College Road, Singapore 169857, Singapore; 6 Global Alliance and External Research, Santen Inc., Emeryville, CA 94608, USA; Najam.sharif@santen.com

**Keywords:** proteasome inhibition, autophagy, retinal degeneration, neuroprotection

## Abstract

We recently demonstrated that chemical proteasome inhibition induced inner retinal degeneration, supporting the pivotal roles of the ubiquitin–proteasome system in retinal structural integrity maintenance. In this study, using beclin1-heterozygous (*Becn1*-Het) mice with autophagic dysfunction, we tested our hypothesis that autophagy could be a compensatory retinal protective mechanism for proteasomal impairment. Despite the reduced number of autophagosome, the ocular tissue morphology and intraocular pressure were normal. Surprisingly, *Becn1*-Het mice experienced the same extent of retinal degeneration as was observed in wild-type mice, following an intravitreal injection of a chemical proteasome inhibitor. Similarly, these mice equally responded to other chemical insults, including endoplasmic reticulum stress inducer, N-methyl-D-aspartate, and lipopolysaccharide. Interestingly, in cultured neuroblastoma cells, we found that the mammalian target of rapamycin-independent autophagy activators, lithium chloride and rilmenidine, rescued these cells against proteasome inhibition-induced death. These results suggest that *Becn1*-mediated autophagy is not an effective intrinsic protective mechanism for retinal damage induced by insults, including impaired proteasomal activity; furthermore, autophagic activation beyond normal levels is required to alleviate the cytotoxic effect of proteasomal inhibition. Further studies are underway to delineate the precise roles of different forms of autophagy, and investigate the effects of their activation in rescuing retinal neurons under various pathological conditions.

## 1. Introduction

The accumulation of toxic protein aggregates is a hallmark feature of many neurodegenerative conditions, such as Alzheimer’s disease, Parkinson’s disease, Huntington’s disease, and amyotrophic lateral sclerosis [1]. The evidence points to a dysfunctional ubiquitin–proteasome system (UPS) to be implicated in the pathogenesis of these diseases [2]. UPS promotes the degradation of most soluble proteins via ubiquitination of target proteins, as well as misfolded and damaged proteins via ubiquitin-independent pathway, preventing the formation of protein aggregates [3,4]. Proteasome activity is decreased in aged neurons and in a plethora of neurodegenerative conditions, leading to the accumulation of aggregated proteins and the formation of ubiquitin-positive inclusion bodies [5]. Proteasome inhibition in animal models, by genetic or pharmacological means, closely mimics the pathology and symptoms of neurodegenerative diseases, such as the aggregation of ubiquitinated proteins, formation of inclusion bodies, and neuronal cell death [6,7,8,9,10]. 

Another major intracellular pathway regulating the clearance of aberrant protein is autophagy, which operates in a closely interlinked manner to the UPS [11]. Autophagy targets large protein complexes and aggregates, and even damaged cellular organelles [12]. The essential role of autophagy in the clearance of neurotoxic protein aggregates in neurodegenerative diseases has been reported in many studies. The genetic deletion of autophagy-related genes in neuronal cells led to neurodegeneration [13,14], whereas activation of autophagy via chemical or genetic means, was shown to enhance the clearance of disease-associated protein aggregates [11]. Thus, these two major protein degradation control systems, the UPS and autophagy, are effective cellular defense mechanisms in neurons and may be potential therapeutic targets for neurodegenerative diseases that are caused by neurotoxic protein accumulation and aggregation. 

As seen in the central nervous system, multiple studies have demonstrated that UPS and autophagy may play significant roles in protein homeostasis in the retina. Retinal proteasome and autophagic activities decline with age, and an increased protein aggregation is found in the aging retina [15,16,17,18]. Impaired proteasome function was reported to induce photoreceptor degeneration [19,20]; conversely, proteasome activation increased photoreceptor survival in an inherited retinal degeneration model [21]. Similarly, autophagy has been implicated in photoreceptor cell death and survival [22,23,24], as well as in retinal pigmented epithelial cell dysfunction in the pathogenesis of age-related macular degeneration [25,26,27]. Recent studies have also linked autophagy to retinal ganglion cell (RGC) death in glaucoma [28]. Despite these efforts, the interplay between UPS and autophagy in the regulation of protein degradation in retinal health and disease is not fully understood at present.

To address this key question, we hypothesized that autophagy could be an innate defense mechanism against retinal degeneration while proteasome function is impaired. We previously reported that the pharmacological inhibition of proteasome in the retina induced an accumulation of poly-ubiquitinated proteins, leading to cell loss in the ganglion cell layer (GCL) [29], which is resistant to an array of pharmacological agents, such as antioxidants and endoplasmic reticulum (ER) stress inhibitors. In this study, we used this chemical proteasome inhibition model in genetically engineered mice with a defect in beclin1 (*Becn1*) gene, a key signaling molecule in the autophagic pathway [30]. They were *Becn1*-heterozygous (*Becn1*-Het) with autophagic dysfunction, which have been used in many earlier studies [31,32,33,34,35]. To our surprise, despite autophagic dysfunction in the retina of these mice, *Becn1*-Het mice and normal wild-type (WT) mice displayed equal vulnerability to retinal degeneration, induced by chemical proteasome inhibition as well as other pharmacological insults. Furthermore, we demonstrated that only the mammalian target of rapamycin (mTOR)-independent autophagy activators could protect against proteasome impairment-induced cytotoxicity in human neuroblastoma cells.

## 2. Results

### 2.1. Intravitreal (IVT) Injection of a Proteasome Inhibitor Induced Inner Retinal Degeneration in Mice

**Figure 1:**
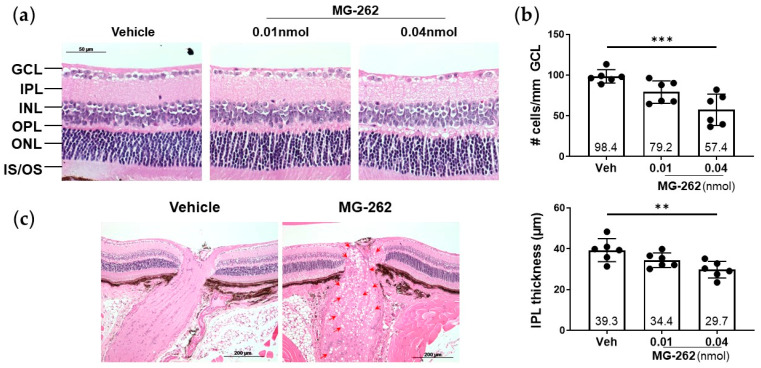
Proteasome inhibitor (MG-262) induced inner retinal degeneration in mice. (**a**) Representative images of hematoxylin and eosin staining of mouse retinae 7 days following intravitreal injection of 0.01 and 0.04 nmol/eye of MG-262, or its vehicle (10% dimethyl sulfoxide in Dulbecco’s phosphate buffered solution or D-PBS). Retinae were cross-sectioned, and their images were taken approximately 300 µm away from the optic nerve head. GCL: ganglion cell layer; IPL: inner plexiform layer; INL: inner nuclear layer; OPL: outer plexiform layer; ONL: outer nuclear layer; IS/OS: photoreceptor inner and outer segments. The scale bar shows 50 µm. (**b**) Quantification of number of cells in the GCL and IPL thickness measurement. Cell counting and thickness measurement were performed on ImageJ. N = 6 eyes from 3 mice per each treatment group. Each value represents the mean ± standard deviation with the mean value indicated at the bottom of each bar. Statistical analysis was performed with one-way analysis of variance, followed by Dunnett’s post hoc test. ** *p* < 0.01 *** *p* < 0.001, vs. vehicle (Veh). (**c**) Representative images of the optic nerve of eyes, injected with 0.01 nmol/eye of MG-262. Vacuolation (red arrow heads) was observed throughout the optic nerve in eyes injected with MG-262. The scale bar shows 200 µm.

To extend our previous finding that in rats intravitreal (IVT) injection of the proteasome inhibitor MG-262 induces inner retinal degeneration [29], we first aimed to reproduce this observation in normal WT mice. Consistent with our previous study, MG-262 (0.01 and 0.04 nmol/eye) induced a severe loss of cells in the GCL, accompanied by inner plexiform layer (IPL) thinning in a dose-dependent manner, while the inner and outer nuclear layers (INL and ONL, respectively) appeared relatively normal 7 days following IVT injection of this agent (Figure 1a,b). Upon MG-262 injection, these retinal morphological changes were associated with the presence of vacuoles in the optic nerve, indicating optic neuropathy (Figure 1c). These findings further increase the value and robustness of chemical proteasome inhibition as a model for inner retinal degeneration in rodents.

### 2.2. Characterization of Ocular Phenotypes of Beclin1-Heterozygous (Becn1-Het) Mice with Reduced Level of Retinal Autophagy

**Figure 2:**
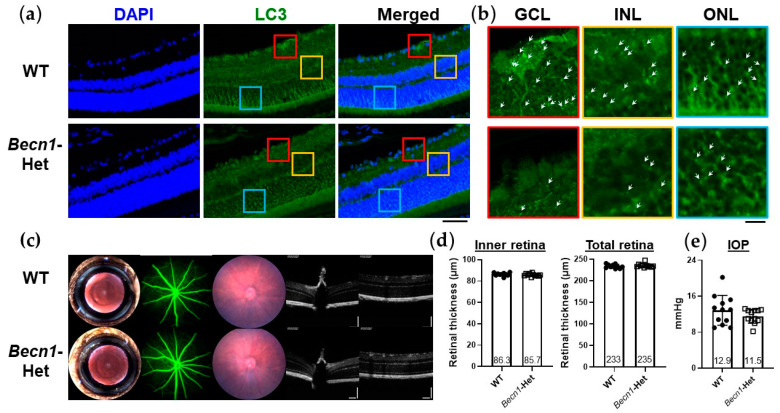
Retinal autophagic dysfunction and ocular phenotypes of beclin1-heterozygous mice (*Becn1*-Het). (**a**) Immunofluorescence staining of LC3 in the retina of wild-type (WT) and *Becn1*-Het mice (each image is representative of 2 eyes). Immunofluorescence staining was performed on 5 µm thick retinal cryosections of WT and *Becn1*-Het mice, and images were acquired with confocal microscopy. Representative merged z-stack images are shown. The scale bar shows 50 µm. (**b**) Enlarged images of LC3 immunostaining in the ganglion cell layer (GCL), inner nuclear layer (INL), and outer nuclear layer (ONL). Red, yellow, and blue colors correspond to the respective area of the images shown in (**a**). White arrow heads indicate visible LC3 punctae. The scale bar shows 10 µm. (**c**) Representative clinical imaging of the eyes of WT and *Becn1*-Het mice, including (from left to right), slit lamp photography, fundus fluorescein angiography, fundus photography, and spectral domain ocular coherence tomography (OCT). (**d**) Inner and total retina thickness was quantified based on the OCT images obtained. Image quantification was performed on ImageJ. The inner retina is defined to include the GCL, IPL (inner plexiform layer), and INL, whereas the total retina includes all retinal layers. N = 10–12 eyes from 5–6 mice per each group. (**e**) Intraocular pression (IOP) measured with rebound tonometer in WT and *Becn1*-Het mice. The same mice shown in (**d**) were used for IOP measurements. Each value represents the mean ± standard deviation with the mean value indicated at the bottom of each bar. Statistical analysis was performed with an unpaired *t*-test.

To test our hypothesis that autophagy could confer retinal protection against impaired proteasome, we characterized retinal phenotypes of *Becn1*-Het mice, being genetically modified mice, with autophagic dysfunction [31,32,33,34] under normal conditions. First, we confirmed a reduced level of autophagy in the retina of these mice using an immunostaining for LC3, a component of the autophagosome (Figure 2a,b). In accordance with previous reports that detected LC3 immunostaining in highly metabolic retinal cell layers [25], we observed LC3 punctae in retinal cell layers, including the GCL, INL, and ONL (Figure 2b). In the *Becn1*-Het mice, LC3 punctae were markedly reduced in all three layers (Figure 2b), confirming the reduced number of autophagosome in these cells. This result further supports the use of *Becn1*-Het mice as an animal model of defective autophagy.

We then determined if any ocular abnormality may be present in *Becn1*-Het mice. As shown in Figure 2c,d, a series of clinical imaging, namely, slit lamp photography, fundus photography, and fundus fluorescent angiography, failed to detect noticeable differences in the morphology of the cornea, the lens, and the retina between WT and *Becn1*-Het mice. Optical coherence tomography further confirmed that the structure of the optic nerve head, as well as the inner and total retinal thickness (Figure 2c,d) of these mice were normal. Additionally, intraocular pressure (IOP) was maintained in the normal range in both groups of mice (Figure 2e). Overall, despite autophagic dysfunction found in the retina of these mice, the eyes of *Becn1*-Het did not display any abnormality in their morphology and structure or IOP under normal conditions.

### 2.3. Becn1-Het and Wild-Type (WT) Mice Possess Equal Vulnerability to Proteasome Inhibition and Endoplasmic Reticulum (ER) Stress

**Figure 3:**
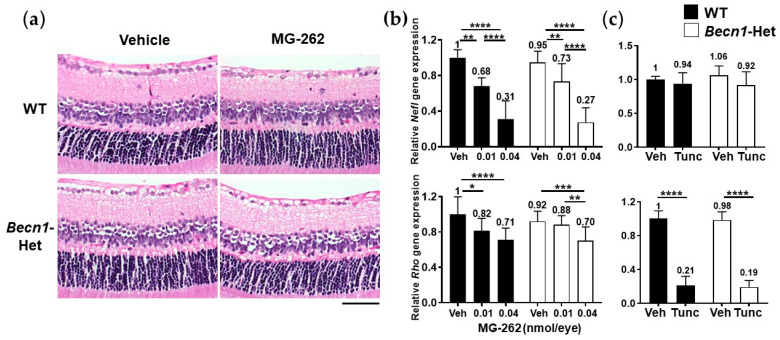
Beclin1-heterozygous mice (*Becn1*-Het) and wild-type (WT) mice are equally sensitive to retinal degeneration, induced by proteasome inhibition and endoplasmic reticulum (ER) stress. Chemicals, or their vehicle, were intravitreally injected into the eyes of WT and *Becn1*-Het mice, and eyes were isolated 7 and 4 days post-injection for histological evaluation (**a**) and gene expression analysis (**b**,**c**), respectively. Quantitative PCR was performed for neurofilament light chain (*Nefl*) and rhodopsin (*Rho*) genes. Each expression value was normalized to glyceraldehyde-3-phosphate dehydrogenase (*Gapdh)*, the housekeeping gene, in individual samples and is shown relative to that obtained in the WT vehicle group. (**a**) Representative images of hematoxylin and eosin-stained retinae, following intravitreal injection of 0.01 nmol/eye MG-262 or its vehicle (10% dimethyl sulfoxide or DMSO in Dulbecco’s phosphate buffered solution or D-PBS). The scale bar shows 50 µm. (**b**) Effects of MG-262 (0.01 and 0.04 nmol/eye) or its vehicle (Veh, 10% DMSO in D-PBS) on *Nefl* and *Rho* gene expression levels in the retina. N = 8 to 16 eyes from 4 to 8 mice per each group. Black and while bars show WT and *Becn1*-Het, respectively. (**c**) Effects of tunicamycin (Tunc, 2.5 µg/eye) or its vehicle (Veh, 25% DMSO in D-PBS) on *Nefl* and *Rho* gene expression levels in the retina. N = 5 to 8 eyes from 4 mice per each group. Each value represents the mean ± standard deviation with the mean value indicated at the top of each bar. For statistical analysis, a two-way analysis of variance was performed, followed by Sidak’s post hoc test. * *p* < 0.05, ** *p* < 0.01, *** *p* < 0.001, **** *p* < 0.0001 for each pair of comparison.

We further examined if autophagic dysfunction in *Becn1*-Het mice could render them more sensitive to inner retinal degeneration caused by proteasome inhibition. As seen above, an IVT injection of MG-262 (0.01 nmol/eye) induced moderate cell loss in the GCL and IPL thinning in both WT and *Becn1*-Het mice (Figure 3a). Qualitatively, a similar degree of inner retinal degeneration upon MG-262 treatment was observed between WT and *Becn1*-Het mice. To quantify these changes more precisely, we used the expression level of genetic cell markers, neurofilament light chain (*Nefl*), and rhodopsin (*Rho*) genes, to reflect the number of RGCs [36] and photoreceptors [37] in the whole retina, respectively. Consistent with our previous study [29], MG-262 (0.01 and 0.04 nmol/eye) reduced *Nefl* expression in a dose-dependent manner in both mouse groups, and the maximum reduction from the respective control level in the vehicle group was approximately 70% (Figure 3b). In contrast, MG-262 reduced the *Rho* expression level by only 30% at the higher dose, compared with the vehicle group. Again, we observed no statistically significant difference in the level of expression of *Nefl* or *Rho* upon MG-262 injection between WT and *Becn1*-Het mice (Figure 3b).

We also tested both animal groups for the retinal effect of tunicamycin, an ER stress inducer that causes dramatic loss of the photoreceptor cells [29,38]. Consistently, IVT injection of tunicamycin induced a dramatic reduction in the expression level of *Rho*, but not *Nefl* gene (Figure 3c). As seen by the effect of MG-262 on the inner retinal degenerative, we did not observe any significant difference in the *Nefl or Rho* level between WT and *Becn1*-Het mice upon injection of tunicamycin (Figure 3c).

Thus, these results suggest that the basal level of autophagic activity does not contribute to the maintenance of the retinal structural integrity under the normal condition, or when protein degradation or folding is impaired following chemical proteasome inhibition or ER stress induction. 

### 2.4. Retinal Degenerative Effects of Other Pharmacological Insults in Becn1-Het Mice

**Figure 4:**
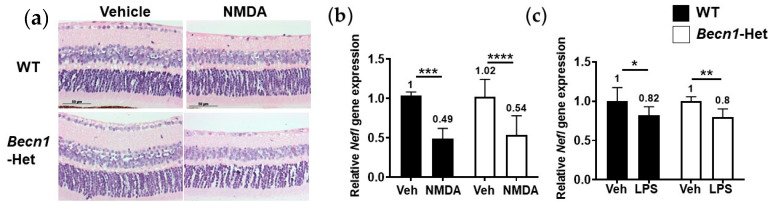
Beclin1-heterozygous mice (*Becn1*-Het) and wild-type (WT) mice are equally sensitive to excitotoxicity and neuroinflammation. Chemicals or their vehicle (Dulbecco’s phosphate buffered solution or D-PBS) were intravitreally injected into the eyes of WT and *Becn1*-Het mice, and eyes were isolated 7 and 4 days post-injection for histological evaluation (**a**) and gene expression analysis (**b**,**c**), respectively. Quantitative PCR was performed for the neurofilament light chain (*Nefl*) gene. Each expression value was normalized to glyceraldehyde-3-phosphate dehydrogenase (*Gapdh)*, the housekeeping gene, in individual samples and is shown relative to that obtained in the WT vehicle group. (**a**) Representative images of hematoxylin and eosin-stained retinae following intravitreal injection of 20 nmol/eye N-methyl-D-aspartate (NMDA) or its vehicle. (**b**) Effects of 20 nmol/eye NMDA or its vehicle on *Nefl* gene expression in the retina. N = 4 to 9 eyes from 2 to 5 mice for each group. (**c**) Effects of 2 µg/eye lipopolysaccharide (LPS) or its vehicle on *Nefl* gene expression in the retina. N = 6 to 9 eyes from 4 to 5 mice for each group. Each value represents the mean ± standard deviation with the mean value indicated at the top of each bar. For statistical analysis, a two-way analysis of variance was performed, followed by Sidak’s post hoc test * *p* < 0.05, ** *p* < 0.01, *** *p* < 0.001, **** *p* < 0.0001 for each pair of comparison.

To extend our findings to other types of retinal degeneration models, we asked if excitotoxicity- and neuroinflammation-induced RGC death could be modified in *Becn1*-Het mice. IVT injection of N-methyl-D-aspartate (NMDA, 20 nmol/eye), an authentic NMDA receptor-coupled channel agonist [38,39], led to cell loss in the GCL and IPL thinning, accompanied by a remarkable reduction in *Nefl* gene expression levels (Figure 4a,b). Similarly, lipopolysaccharide (LPS, 2 µg/eye), a pro-inflammatory substance [40], caused a mild but statistically significant reduction in *Nefl* gene expression. Similar to the retinal degeneration induced by MG-262 and tunicamycin IVT injection, we did not observe any statistically significant difference in the retinal structural changes and reduction in *Nefl* gene expression upon IVT injection of NMDA or LPS between WT and *Becn1*-Het mice (Figure 4b,c).

Altogether, it is likely that *Becn1*-mediated autophagy may not compensate for retinal damage induced by an array of deleterious insults, including chemical proteasome inhibition, ER stress, excitotoxicity, and inflammation in mice. 

### 2.5. Specific Autophagy Activators Could Protect against MG-262-Induced Cytotoxicity

**Figure 5:**
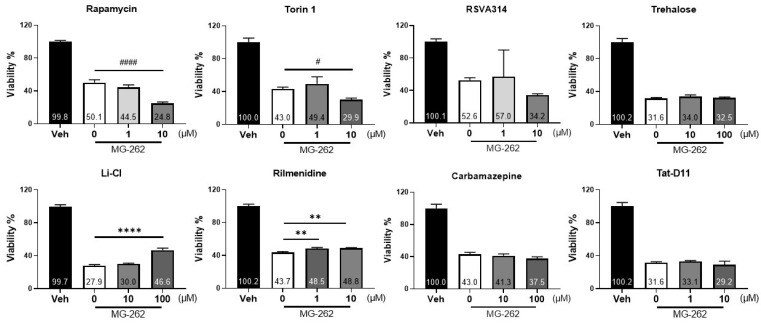
Autophagy activators could alleviate MG-262-induced cytotoxicity. Human neuroblastoma cells, SH-SY5Y, were treated with a series of different autophagy activators, as indicated, 1 h prior to treatment with 0.2 µM MG-262 or its vehicle (0.1% dimethyl sulfoxide in Dulbecco’s Modified Eagle’s Medium) for 24 h, and cell viability was measured by WST-1 assay. All experiments were performed in triplicate and each value is shown as a percentage of that obtained in the vehicle group. Statistical test was performed with one-way analysis of variance, followed by Dunnett’s post hoc test. Each value represents the mean ± standard deviation with the mean value indicated at the bottom of each bar. ** *p* < 0.01, **** *p* < 0.0001, statistically significant increase in cell viability compared with the respective vehicle group; # *p* < 0.05; #### *p* < 0.0001, statistically significant reduction in cell viability compared with the respective vehicle group.

Finally, we tested the possibility that extrinsic activation of autophagy may offer additional protection against cellular damage induced by proteasomal inhibition because the normal level of autophagic activity may not be sufficient to protect neuronal cells. To test this possibility, we chose human neuroblastoma cells, SH-SY5Y, often used as surrogate cells for primary brain neurons [41] because of the less complex and smaller data variabilities, compared with in vivo experiments. We examined the effects of a series of mTOR-dependent (rapamycin, Torin-1, RSVA314 and Tat-D11) [42,43,44] and independent autophagy activators (trehalose, carbamazepine, lithium chloride, and rilmenidine) [45] on MG-262-induced neuronal cell death. As shown in Figure 5, MG-262 consistently induced cell death in SH-SY5Y cells throughout the experiments. Among all tested compounds, only two mTOR-independent autophagy activators, lithium chloride and rilmenidine, were found to increase cell viability when treated prior to MG-262 exposure. The maximum cytoprotective effects of lithium chloride was greater than that of rilmenidine, although both effects were statistically significant. Regardless of mTOR-dependent or independent activation, the other chemicals (rapamycin, Torin-1, RSVA314, Tat-D11, carbamazepine or trehalose) did not rescue these cells. Interestingly, in our experimental setting, the classic mTOR inhibitors, rapamycin and Torin-1, exacerbated MG-262-induced cytotoxicity in a statistically significant manner. These data support the notion that mTOR-independent autophagic activation may play a cytoprotective role against cell death induced by chemical proteasome inhibition in vitro.

## 3. Discussion

The key finding of this study is that autophagic dysfunction did not modify inner retinal degeneration induced by chemical proteasome inhibition in *Becn1*-Het mice. To our knowledge, this is the first report studying the potential roles of autophagy in the retinal structural integrity, while proteasome function is impaired. Given the proposed retinal protective roles of autophagy in earlier studies [46,47,48,49], we hypothesized that autophagy could also be a compensatory mechanism to overcome toxic protein accumulation in the event of proteasome impairment in the retina, and that autophagic dysfunction might exacerbate the degree of retinal degeneration induced by chemical proteasome inhibition. Unexpectedly, this hypothesis was not supported by the findings that *Becn1*-Het and WT mice displayed equal vulnerability to retinal damage induced by chemical proteasome inhibition as well as other pharmacological insults, including ER stress, excitotoxicity, and neuroinflammation. However, we found that mTOR-independent autophagy activators, lithium chloride and rilmenidine, could significantly alleviate chemical proteasome inhibition-induced cell death in vitro. These results suggest that although the intrinsic autophagic activity does not play a major role in retinal cell survival, the extrinsic stimulation of autophagy promotes cytoprotection against cell death caused by proteasome impairment in vitro. 

We previously reported that chemical proteasome inhibition could be used as a novel animal model for inner retinal degeneration in rats. IVT administration of MG-262 (0.1 nmol/eye), a potent and selective proteasome inhibitor [50], reduces retinal proteasome activity, resulting in the accumulation of ubiquitinated proteins in the rat retina [29]. In this study, we chose two doses of MG-262 (0.01 and 0.04 nmol/eye), which correspond to the estimated vitreous concentration (1.7 µM) following MG-262 injection of the tested dose in the rat eye, given the difference in the vitreous volume between the rat and mouse eyes (60 vs. 10 µL). We consistently replicated the previous findings that IVT injection of the proteasome inhibitor MG-262 caused a severe loss of cells in the GCL and IPL thinning, while leaving the photoreceptor layer relatively intact in normal mice. This further demonstrates the robustness of this model. We combined this model with *Becn1*-Het knockout in mice. *Becn1* is an evolutionary conserved protein, which plays a key role in autophagic vesicle nucleation, together with class III phosphatidylinositol 3-kinase (PI3K) complex [30]. Homozygous deletion of *Becn1* is embryonically lethal, while *Becn1* gene haploinsufficiency in the heterozygote mice was shown to promote tumorigenesis [31,32] and neurodegeneration [33], via impaired autophagy. *Becn1*-Het mice are one of the most popular animal models used to study the implications of autophagic dysfunction [31], which has been demonstrated in various tissues, including muscle, bronchial epithelial cells, germinal center B cells [31], heart aorta [34], and hippocampal neurons [33]. In contrast to these earlier studies and to our initial hypothesis, we found that *Becn1*-Het mice did not show any ocular abnormalities under normal conditions, as seen in an earlier study [35]. More importantly, *Becn1*-Het knockout did not affect the susceptibility of these mice to various pharmacological insults, including proteasome inhibition. Therefore, it is reasonable to conclude that autophagic dysfunction, due to *Becn1* haploinsufficiency, may not contribute to the maintenance of the retinal structural integrity while proteasome function is impaired. However, we cannot exclude the possibility that the complete absence of autophagy in *Becn1* homozygous knockout in the retina may enable RGCs to be more sensitive to these insults. In fact, such a scenario can be seen in an earlier study, demonstrating that conditional knockout of *Atg5*, another autophagy regulatory gene, in macrophages induced inflammasome activation and promoted atherosclerosis progression; however, the modest disruption of autophagy in *Becn1*-Het mice showed no phenotype [34]. Further studies using *Becn1* conditional knockout in the retina are underway, to determine whether *Becn1* homozygous deletion may have a different impact on inner retinal degeneration induced by chemical proteasome inhibition.

Although the interplay between UPS and autophagy in the retina is largely unknown, an interesting concept has been proposed in recent studies using *Rho* mutant mice [51,52]. These investigators demonstrated that an activated autophagic function by a missense mutation in *Rho* gene and an mTOR inhibitor exacerbated retinal degeneration, whereas reduced autophagic activity by retinal specific deletion of *Atg5* and the autophagy inhibitor hydroxychloroquine protected photoreceptors from *Rho* mutation-induced degeneration. Furthermore, they found that increased ER stress mediated overactivation of autophagy in response to *Rho* mutation, leading to a reduced UPS function and thereby photoreceptor degeneration. These findings suggest that a balance between UPS and autophagy activities is critical for photoreceptor survival, and either inhibition of autophagy or activation of UPS can be a potential therapeutic approach for retinal diseases, including photoreceptor degeneration. Their findings and concept contradict the proposed protective roles of autophagy in the retina in earlier studies [46,47,48,53]. However, a significant number of studies have also reported consistent results, where the inhibition of autophagy could be a retinal protective [49,54,55,56]. For example, the autophagy inhibitor 3-methyl adenine was reported to suppress RGC death in a retinal ischemia model [56]. In the current study, we found that reduced autophagy activity did not alter inner retinal degeneration indued by proteasome inhibition in *Becn1*-Het mice. Taking into account the above proposed concept of a balance between UPS and autophagy, one possible interpretation of our findings is that autophagy may not induce further cell death when UPS function is already eliminated, following chemical proteasome inhibition. However, this possibility is unlikely because autophagic dysfunction in *Becn1*-Het mice had no impact on inner retinal degeneration induced by ER stress, excitotoxicity, and neuroinflammation. Therefore, autophagy may not play significant retinal protective or detrimental roles against inner retinal degeneration induced by a wide variety of chemical insults.

While autophagy may not be an intrinsic protective mechanism, it is possible that extrinsic activation of autophagy may provide neuroprotection while proteasome activity is reduced. To eliminate the complexities associated with in vivo studies, including the pharmacokinetic behavior of tested compounds, and minimized data variabilities among individual animals, we used cultured neuronal cells, SH-SY5Y, instead of whole animals. SH-SY5Y cells are well established as model neurons to study neuroprotection and neurodegenerative mechanisms [41,57,58], and are widely used to study autophagy and UPS [59,60,61,62]. Interestingly, we found that lithium chloride and rilmenidine promoted cell protection, whereas rapamycin and Torin-1 exacerbated cell death in SH-SY5Y neuroblastoma cells exposed to proteasome inhibition. Rapamycin and Torin-1 are inhibitors of mTOR [42], a sensor of nutrient availability and recipient of growth factor signaling [63]. mTOR negatively regulates the formation of the ULK complex via phosphorylation of ULK1/2 [64,65], which is the initial step of autophagic vesicle nucleation. Following this initial step, *Becn1* regulates the formation of another complex called the PI3K complex. In addition to this, autophagy is regulated by mTOR-independent pathways that involve other intracellular signaling molecules, including inositol and cyclic adenosine monophosphate (cAMP) [66]. Rilmenidine reduces cAMP via stimulation of imidazoline-1 receptors [67], whereas lithium chloride lowers intracellular inositol levels via the inhibition of inositol monophosphatase 1 [68,69]. Reduced levels in both inositol and cAMP lead to autophagy activation in a mTOR-independent manner [68,69], although lithium chloride has also been reported to contribute to the mTOR-dependent pathway via the inhibition of glycogen synthase kinase 3 beta [70]. Although the exact molecular mechanisms are unclear, extrinsic activation of mTOR-independent autophagic pathways may overcome cellular stress caused by chemical proteasome inhibition, while that of mTOR-dependent pathways may enhance this stress, resulting in the opposite outcomes in cell survival and death in neuroblastoma cells. Given the significance of autophagy in the pathogenesis of neurodegenerative diseases, in depth in vitro and in vivo analyses of the molecular mechanisms of action for autophagy activators and inhibitors is necessary to identify potential new drug candidates for the treatment of relevant diseases, as described elsewhere [71,72]. 

In conclusion, the present study demonstrates that *Becn1*-Het knockout does not rescue or sensitize inner retinal cells against degeneration induced by chemical proteasome inhibition in mice. Furthermore, we provide evidence demonstrating that mTOR-independent autophagy activators may be protective in neuroblastoma cells and thus can be regarded as neuroprotective agents. These results suggest that intrinsic autophagy activities are not sufficient in operating to protect inner retinal cells as a compensatory mechanism for impaired proteasome function; furthermore, extrinsic autophagy activation via mTOR-independent pathways still promotes neuroprotection while proteasome function is compromised in vitro. Thus, our current study further supports the importance of UPS function in the maintenance of the retinal structural integrity, as demonstrated in our previous study [29], and provides some insights into the interplay between UPS and autophagy in the retina. A thorough study characterizing the effect of autophagy and proteasome modulators on their protein expression levels and activities may uncover novel crosstalk regulation between the two pathways in the retina. Such a study may also warrant further development of mTOR-independent autophagy activator as a therapeutic modality for retinal neurodegenerative diseases associated with proteasome dysfunction [73,74]. 

## 4. Materials and Methods

### 4.1. Chemicals

MG-262, NMDA, and rapamycin were purchased from Enzo Life Sciences, Inc. (Farmingdale, NY, USA). LPS was purchased from Invitrogen (San Diego, CA, USA). Torin-1 and rilmenidine hemifumarate were purchased from Tocris Bioscience (Avonmouth, Bristol, UK). Tat-D11 (Tat- *Becn1* D11) was purchased from Novus Biologicals (Littleton, CO, USA). All other chemicals were obtained from Sigma Aldrich (St Louis, MO, USA). All chemicals were dissolved in dimethyl sulfoxide (Thermo Fisher Scientific Inc., Waltham, MA, USA) or distilled water to prepare a stock solution, and diluted with Dulbecco’s phosphate buffered solution (D-PBS; from Life Technologies Corp, Carlsbad, CA, USA) to reach the final indicated concentration.

### 4.2. Mice

*Becn1*-Het mice (B6.129X1-*Becn1*^tm1Blev^/J) were purchased from The Jackson Laboratory (Bar Harbor, ME, USA). They were bred and housed in the animal facility at SingHealth Experimental Medicine Centre, where they were maintained under a 12 h light (~10 lux)/12 h dark cycle and provided food and water ad libitum. Genotyping was performed according to the protocol provided by The Jackson Laboratory. All animal experimental procedures were approved and performed in accordance with Institutional Animal Care and Use Committee (IACUC) guidelines of SingHealth Singapore and the Association for Research in Vision or Ophthalmology (ARVO) statement for the Use of Animals in Ophthalmic and Vision Research.

### 4.3. IVT Injection

The procedure for IVT injection has been described previously [29,75,76]. Briefly, prior to injection animals were anesthetized with 150 mg/kg body weight (BW) ketamine hydrochloride (Parnell Laboratories, New South Wales, Australia) and 10 mg/kg BW xylazine hydrochloride (Troy Laboratories, New South Wales, Australia) and their pupils were dilated with a topical application of 1% tropicamide and 2.5% phenylephrine hydrochloride (Alcon Laboratories Inc., Fort Worth, TX, USA). For the injection, a 1 µL aliquot of solution containing each chemical was injected into the vitreous body of both eyes of each mouse using a Nanofil microsyringe with a 33G needle (World Precision Instruments Inc., Sarasota, FL, USA), under an ocular surgery microscope (Leica Microsystems GmbH, Wetzlar, Germany). A bilateral approach was taken to minimize the number of animals used in this study. All procedures were performed carefully to avoid injuring the lens or retina. Four or seven days following injections, the eyes were collected and subjected to further analysis as described below. Eyes with bleeding or inflammation caused by technical errors were excluded from analysis.

### 4.4. Intraocular Pressure (IOP) Measurement and Imaging

Animals were anaesthetized with ketamine and xylazine in the same way as described in the above section. IOP was measured with an Icare TONOLAB rebound tonometer (Icare Finland Oy, Vantaa, Finland), approximately five minutes after animals were sedated. IOP was recorded between 9 AM and 11 AM in the room lighting at approximately 700 lux. The average of 5 consecutive measurements per eye was taken each time as the representative value.

Digital color fundus photographs, spectral domain OCT images, and fundus fluorescent angiography (FFA) images were taken using the Micron IV retinal imaging microscope (Phoenix Research Laboratories, Pleasanton, CA, USA), centered on the optic nerve head and in all four quadrants. Fundus imaging and fluorescein angiography were performed following pupil dilation, as described above. FFA was performed after intraperitoneal injections of 1% sodium fluorescein dye (Akorn Inc., Lake Forest, IL, USA), diluted in sterile saline and given at a dose of 0.1 mL/10 g BW. FFA images were taken approximately 30 s after the fluorescein injection.

### 4.5. Histological Evaluation and Immunostaining

Eyes for histology were fixed in 2.5% glutaraldehyde (Agar Scientific Ltd., Essex, UK) and 10% formaldehyde (Sigma Aldrich, St Louis, MO, USA) for 24 h at room temperature with gentle shaking. The fixed eyes were embedded in paraffin and the retinae were cross-sectioned with 3 µm thickness through the optic disk. Eight cross sections were automatically stained with hematoxylin and eosin (Leica Microsystems GmbH, Wetzlar, Germany) and three sections per an eye were randomly chosen for quantitative measurements. Images were recorded under a brightfield microscope (Axioplan2, Carl Zeiss GmbH, Oberkochen, Germany). Approximately 400 µm width of the retina, starting at 300 µm from the center of the optic disk, was analyzed and the number of cells in the GCL and IPL thickness were measured by ImageJ (version1.53; NIH, Bethesda, MD, USA). The values obtained in three sections were averaged as the representative value for each eye.

For immunostaining, an antigen retrieval step was performed using a high pH target retrieval solution (Agilent Dako, Santa Clara, CA, USA). LC3 immunostaining was performed using a LC3 antibody (Sigma Aldrich, St Louis, MO, USA) in an antibody dilution buffer (2% BSA, 0.1% Triton in D- PBS) for overnight at 4 °C, followed by labelling with a secondary antibody conjugated to Alexa Fluor-488 (Thermo Fisher Scientific Inc., Waltham, MA, USA). Nuclei were labelled by incubating the sections in DAPI-containing D-PBS for 10 min at room temperature, before mounting with Prolong Diamond antifade (all from Life Technologies Corp, Carlsbad, CA, USA). Confocal images were taken with Leica SP8 microscopy (Leica Microsystems GmbH, Wetzlar, Germany) and analysed with Leica Application Suite X (LAS X) software (version 3.7.4; Leica Microsystems GmbH, Wetzlar, Germany). 

### 4.6. Quantitative PCR

According to the procedures described previously [29,75,76] and the manufacturer’s instructions, a quantitative PCR was performed. Briefly, total RNA was extracted using a RNeasy Lipid Tissue Mini kit (Qiagen, Hilden, Germany) and cDNA was synthesized using 0.4 µg of total RNA for each retina in a 20 µL mixture containing Superscipt Enzyme Mix, oligo-DT primers, and random hexamers (Life Technologies Corp, Carlsbad, CA, USA). The resultant cDNA was then diluted 20 times, and a 2 µL aliquot was added into a mixture containing SsoAdvanced Universal SYBR Green Supermix (Biorad, Hercules, CA, USA) and primer mix (Integrated DNA Technologies, Coralville, IA, USA). The sequences of forward and reverse primers used were listed in Table 1. Quantitative PCR was performed using Quantstudio 7 Flex Real-Time Systems (Thermo Fisher Scientific Inc., Waltham, MA, USA) under the cycling condition at 50 °C for 2 min, 95 °C for 10 min, followed by 40 cycles at 94 °C for 1 min and 60 °C for 1 min. Threshold cycle time (Ct) values were determined using the Quantstudio software (Thermo Fisher Scientific Inc., Waltham, MA, USA). Gene expression levels of *Nefl* relative to that of glyceraldehyde-3-phosphate dehydrogenase (*Gapdh)* were determined by interpolating their Ct values to a standard curve (prepared with increasing amounts of cDNA derived from a 1:1 mixture of total RNA isolated from mouse brain and retina) and were further normalized to the respective control (vehicle group).

### 4.7. Cell Culture and Viability Assay

SH-SY5Y human neuroblastoma cells were obtained from ATCC (Manassas, VA, USA) and cultured in accordance with the supplier’s instruction, in a culture media containing Dulbecco’s Modified Eagle’s Medium, F12 medium, 2 mM L-Glutamine, 10% fetal bovine serum, 0.1 mM non-essential amino acids, 1 mM sodium pyruvate, 1.5 mg/mL sodium bicarbonate, and 100 U/mL penicillin–streptomycin (all from Life Technologies Corp, Carlsbad, CA, USA). 

For viability assay, 10,000 cells were seeded onto a 96 well plate with collagen coating, and chemical treatments were performed 24 h after seeding as indicated. Autophagy activators were added 1 h prior to the addition of MG-262. Cell viability was then measured 24 h after treatment using a WST-1 assay kit (Abcam, Cambridge, UK) according to the manufacturer’s protocol. Absorbance value was measured with Infinite M200 microplate reader (Tecan, Männedorf, Zürich, Switzerland).

### 4.8. Statistical Analysis

Each value represents the mean ± standard deviation. For statistical analysis, an unpaired *t*-test, one-way, or two-way analysis of variance was performed and followed by post hoc analysis, as indicated in the figure legends. Differences were assumed to be statistically significant when *p* < 0.05. All statistical analyses were performed with GraphPad Prism software version 9.0.2 (GraphPad, San Diego, CA, USA).

## Figures and Tables

**Table 1:** Primer sequences used in the quantitative PCR study in 5′ to 3′ direction.

Gene	Forward	Reverse
neurofilament light chain (*Nefl*)	CCGTACTTTTCGACCTCCTACA	CTTGTGTGCGGATAGACTTGAG
rhodopsin (*Rho*)	CCCTTCTCCAACGTCACAGG	TGAGGAAGTTGATGGGGAAGC
glyceraldehyde-3-hosphate dehydrogenase (*Gapdh)* ^1^	TGGCCTTCCGTGTTCCTAC	GAGTTGCTGTTGAAGTCGCA

^1^ Housekeeping gene.

## Data Availability

The data presented in this study are available on request from the corresponding author.

## References

[1] Taylor J.P., Hardy J., Fischbeck K.H. (2002). Toxic Proteins in Neurodegenerative Disease. Science.

[2] Dantuma N.P., Bott L.C. (2014). The ubiquitin-proteasome system in neurodegenerative diseases: Precipitating factor, yet part of the solution. Front. Mol. Neurosci..

[3] Bustamante H.A., Gonzalez A.E., Cerda-Troncoso C., Shaugnessy R., Otth C., Soza A., Burgos P.V. (2018). Interplay between the autophagy-lysosomal pathway and the ubiquitin-proteasome system: A target for therapeutic development in Alzheimer’s disease. Front. Cell. Neurosci..

[4] Raynes R., Pomatto L.C., Davies K.J. (2016). Degradation of oxidized proteins by the proteasome: Distinguishing between the 20S, 26S, and immunoproteasome proteolytic pathways. Mol. Asp. Med..

[5] Saez I., Vilchez D. (2014). The Mechanistic Links Between Proteasome Activity, Aging and Age-related Diseases. Curr. Genom..

[6] McNaught K.S.P., Björklund L.M., Belizaire R., Isacson O., Jenner P., Olanow C.W. (2002). Proteasome inhibition causes nigral degeneration with inclusion bodies in rats. NeuroReport.

[7] McNaught K.S.P., Belizaire R., Jenner P., Olanow C., Isacson O. (2002). Selective loss of 20S proteasome α-subunits in the substantia nigra pars compacta in Parkinson’s disease. Neurosci. Lett..

[8] Xie W., Li X., Li C., Zhu W., Jankovic J., Le W. (2010). Proteasome inhibition modeling nigral neuron degeneration in Parkinson’s disease. J. Neurochem..

[9] Bedford L., Hay D., Devoy A., Paine S., Powe D.G., Seth R., Gray T., Topham I., Fone K., Rezvani N. (2008). Depletion of 26S proteasomes in mouse brain neurons causes neurodegeneration and Lewy-like inclusions resembling human pale bodies. J. Neurosci..

[10] Li Z., Arnaud L., Rockwell P., Figueiredo-Pereira M.E. (2004). A single amino acid substitution in a proteasome subunit triggers aggregation of ubiquitinated proteins in stressed neuronal cells. J. Neurochem..

[11] Lim J., Yue Z. (2015). Neuronal Aggregates: Formation, Clearance, and Spreading. Dev. Cell.

[12] Komatsu M., Ueno T., Waguri S., Uchiyama Y., Kominami E., Tanaka K. (2007). Constitutive autophagy: Vital role in clearance of unfavorable proteins in neurons. Cell Death Differ..

[13] Hara T., Nakamura K., Matsui M., Yamamoto A., Nakahara Y., Suzuki-Migishima R., Yokoyama M., Mishima K., Saito I., Okano H. (2006). Suppression of basal autophagy in neural cells causes neurodegenerative disease in mice. Nature.

[14] Komatsu M., Waguri S., Chiba T., Murata S., Iwata J.-I., Tanida I., Ueno T., Koike M., Uchiyama Y., Kominami E. (2006). Loss of autophagy in the central nervous system causes neurodegeneration in mice. Nature.

[15] Leger F., Fernagut P.-O., Canron M.-H., Léoni S., Vital C., Tison F., Bezard E., Vital A. (2011). Protein Aggregation in the Aging Retina. J. Neuropathol. Exp. Neurol..

[16] Kapphahn R.J., Bigelow E.J., Ferrington D.A. (2007). Age-dependent inhibition of proteasome chymotrypsin-like activity in the retina. Exp. Eye Res..

[17] Louie J.L., Kapphahn R.J., Ferrington D.A. (2002). Proteasome function and protein oxidation in the aged retina. Exp. Eye Res..

[18] Rodríguez-Muela N., Koga H., García-Ledo L., De La Villa P., De La Rosa E.J., Cuervo A.M., Boya P. (2013). Balance between autophagic pathways preserves retinal homeostasis. Aging Cell.

[19] Lobanova E., Finkelstein S., Skiba N.P., Arshavsky V.Y. (2013). Proteasome overload is a common stress factor in multiple forms of inherited retinal degeneration. Proc. Natl. Acad. Sci. USA.

[20] Ando R., Noda K., Tomaru U., Kamoshita M., Ozawa Y., Notomi S., Hisatomi T., Noda M., Kanda A., Ishibashi T. (2014). Decreased Proteasomal Activity Causes Photoreceptor Degeneration in Mice. Investig. Opthalmol. Vis. Sci..

[21] Lobanova E., Finkelstein S., Li J., Travis A.M., Hao Y., Klingeborn M., Skiba N.P., Deshaies R.J., Arshavsky V.Y. (2018). Increased proteasomal activity supports photoreceptor survival in inherited retinal degeneration. Nat. Commun..

[22] Besirli C., Chinskey N.D., Zheng Q.-D., Zacks D.N. (2011). Autophagy Activation in the Injured Photoreceptor Inhibits Fas-Mediated Apoptosis. Investig. Opthalmol. Vis. Sci..

[23] Kunchithapautham K., Rohrer B. (2007). Apoptosis and Autophagy in Photoreceptors Exposed to Oxidative Stress. Autophagy.

[24] Rodríiguez-Muela N., Hernández-Pinto A.M., Serrano-Puebla A., Garcíaledo L., Latorre S., de la Rosa E.J., Boya P. (2015). Lysosomal membrane permeabilization and autophagy blockade contribute to photoreceptor cell death in a mouse model of retinitis pigmentosa. Cell Death Differ..

[25] Mitter S.K., Rao H.V., Qi X., Cai J., Sugrue A., Dunn W.A., Grant M.B., Boulton M.E. (2011). Autophagy in the Retina: A Potential Role in Age-Related Macular Degeneration. Adv. Exp. Med. Biol..

[26] Mitter S.K., Song C., Qi X., Mao H., Rao H., Akin D., Lewin A., Grant M., Dunn W., Ding J. (2014). Dysregulated autophagy in the RPE is associated with increased susceptibility to oxidative stress and AMD. Autophagy.

[27] Golestaneh N., Chu Y., Xiao Y.-Y., Stoleru G.L., Theos A.C. (2018). Dysfunctional autophagy in RPE, a contributing factor in age-related macular degeneration. Cell Death Dis..

[28] Fernández-Albarral J.A. (2021). The Role of Autophagy in Eye Diseases. Life.

[29] Kageyama M., Ota T., Sasaoka M., Katsuta O., Shinomiya K. (2019). Chemical proteasome inhibition as a novel animal model of inner retinal degeneration in rats. PLoS ONE.

[30] Kihara A., Kabeya Y., Ohsumi Y., Yoshimori Y. (2001). Beclin–phosphatidylinositol 3-kinase complex functions at the trans-Golgi network. EMBO Rep..

[31] Qu X., Yu J., Bhagat G., Furuya N., Hibshoosh H., Troxel A., Rosen J., Eskelinen E.-L., Mizushima N., Ohsumi Y. (2003). Promotion of tumorigenesis by heterozygous disruption of the beclin 1 autophagy gene. J. Clin. Investig..

[32] Yue Z., Jin S., Yang C., Levine A.J., Heintz N. (2003). Beclin 1, an autophagy gene essential for early embryonic development, is a haploinsufficient tumor suppressor. Proc. Natl. Acad. Sci. USA.

[33] Pickford F., Masliah E., Britschgi M., Lucin K., Narasimnah R., Jaeger P.A., Small S., Spencer B., Rockenstein B., Levine B. (2008). The autophagy-related protein beclin 1 shows reduced expression in early Alzheimer disease and regulates amyloid β accumulation in mice. J. Clin. Investig..

[34] Razani B., Feng C., Coleman T., Emanuel R., Wen H., Hwang S., Ting J.P., Virgin H., Kastan M.B., Semenkovich C.F. (2012). Autophagy Links Inflammasomes to Atherosclerotic Progression. Cell Metab..

[35] Chen Y., Sawada O., Kohno H., Le Y.-Z., Subauste C., Maeda T., Maeda A. (2013). Autophagy Protects the Retina from Light-induced Degeneration. J. Biol. Chem..

[36] Soto I., Oglesby E., Buckingham B.P., Son J.L., Roberson E.D.O., Steele M.R., Inman D.M., Vetter M.L., Horner P.J., Marsh-Armstrong N. (2008). Retinal Ganglion Cells Downregulate Gene Expression and Lose Their Axons within the Optic Nerve Head in a Mouse Glaucoma Model. J. Neurosci..

[37] Pan Y., Luna R.I.M.-D., Lou C.-H., Nekkalapudi S., Kelly L.E., Sater A.K., El-Hodiri H.M. (2010). Regulation of photoreceptor gene expression by the retinal homeobox (Rx) gene product. Dev. Biol..

[38] Camacho A., Massieu L. (2006). Role of glutamate transporters in the clearance and release of glutamate during ischemia and its relation to neuronal death. Arch. Med Res..

[39] Manev H., Favaron M., Guidotti A., Costa E. (1989). Delayed increase of Ca2^+^ influx elicited by glutamate: Role in neuronal death. Mol. Pharmacol..

[40] Jang S., Lee J.-H., Choi K.-R., Kim N., Yoo H.-S., Oh S. (2006). Cytochemical Alterations in the Rat Retina by LPS Administration. Neurochem. Res..

[41] Xicoy H., Wieringa B., Martens G.J.M. (2017). The SH-SY5Y cell line in Parkinson’s disease research: A systematic review. Mol. Neurodegener..

[42] Kim Y.C., Guan K.-L. (2015). mTOR: A pharmacologic target for autophagy regulation. J. Clin. Investig..

[43] Shoji-Kawata S., Sumpter R., Leveno M., Campbell G.R., Zou Z., Kinch L., Wilkins A.D., Sun Q., Pallauf K., MacDuff D. (2013). Identification of a candidate therapeutic autophagy-inducing peptide. Nat. Cell Biol..

[44] Vingtdeux V., Chandakkar P., Zhao H., d’Abramo C., Davies P., Marambaud P. (2011). Novel synthetic small-molecule activators of AMPK as enhancers of autophagy and amyloid-β peptide degradation. FASEB J..

[45] Sarkar S., Ravikumar B., Floto R.A., Rubinsztein D.C. (2009). Rapamycin and mTOR-independent autophagy inducers ameliorate toxicity of polyglutamine-expanded huntingtin and related proteinopathies. Cell Death Differ..

[46] Bell K., Rosingol I., Sierra-Filardi E., Schmelter C., Grus F., Boya P. (2020). Age related RGC vulnerability in an autophagy deficient mouse model. Investig. Ophthalmol. Vis. Sci..

[47] Rodríguez-Muela N., Germain F., Mariño G., Fitze P.S., Boya P. (2011). Autophagy promotes survival of retinal ganglion cells after optic nerve axotomy in mice. Cell Death Differ..

[48] Russo R., Varano G.P., Adornetto A., Nazio F., Tettamanti G., Girardello R., Cianfanelli V., Cavaliere F., Morrone L.A., Corasaniti M.T. (2018). Rapamycin and fasting sustain autophagy response activated by ischemia/reperfusion injury and promote retinal ganglion cell survival. Cell Death Dis..

[49] Park K.K., Liu K., Hu Y., Smith P.D., Wang C., Cai B., Xu B., Connolly L., Kramvis I., Sahin M. (2008). Promoting Axon Regeneration in the Adult CNS by Modulation of the PTEN/mTOR Pathway. Science.

[50] Kisselev A.F., Goldberg A.L. (2001). Proteasome inhibitors: From research tools to drug candidates. Chem. Biol..

[51] Yao J., Qiu Y., Frontera E., Jia L., Khan N.W., Klionsky D.J., Ferguson T.A., Thompson D.A., Zacks D.N. (2018). Inhibiting autophagy reduces retinal degeneration caused by protein misfolding. Autophagy.

[52] Qiu Y., Yao J., Jia L., Thompson D.A., Zacks D.N. (2019). Shifting the balance of autophagy and proteasome activation reduces proteotoxic cell death: A novel therapeutic approach for restoring photoreceptor homeostasis. Cell Death Dis..

[53] Su W., Li Z., Jia Y., Zhuo Y. (2014). Rapamycin Is Neuroprotective in a Rat Chronic Hypertensive Glaucoma Model. PLoS ONE.

[54] Lim J.-H.A., Stafford B.K., Nguyen B.K.S.P.L., Lien J.-H.A.L.B.V., Wang C., Zukor K., He C.W.Z., Huberman A.D. (2016). Neural activity promotes long-distance, target-specific regeneration of adult retinal axons. Nat. Neurosci..

[55] Chen G., Tang L., Wei W., Li Z., Li Y., Duan X., Chen H. (2016). mTOR regulates neuroprotective effect of immunized CD4^+^Foxp3^+^ T cells in optic nerve ischemia. Sci. Rep..

[56] Piras A., Gianetto D., Conte D., Bosone A., Vercelli A. (2011). Activation of Autophagy in a Rat Model of Retinal Ischemia following High Intraocular Pressure. PLoS ONE.

[57] Kovalevich J., Langford D. (2013). Considerations for the Use of SH-SY5Y Neuroblastoma Cells in Neurobiology.

[58] Bell M., Zempel H. (2021). SH-SY5Y-derived neurons: A human neuronal model system for investigating TAU sorting and neuronal subtype-specific TAU vulnerability. Rev. Neurosci..

[59] Shamoto-Nagai M., Maruyama W., Kato Y., Isobe K.-i., Tanaka M., Naoi M., Osawa T. (2003). An inhibitor of mitochondrial complex I, rotenone, inactivates proteasome by oxidative modification and induces aggregation of oxidized proteins in SH-SY5Y cells. J. Neurosci. Res..

[60] Cheng B., Maffi S.K., Martinez A.A., Acosta Y.P.V., Morales L.D., Roberts J.L. (2011). Insulin-like growth factor-I mediates neuroprotection in proteasome inhibition-induced cytotoxicity in SH-SY5Y cells. Mol. Cell. Neurosci..

[61] Srinivasan V., Bruelle C., Scifo E., Pham D.D., Soliymani R., Lalowski M., Lindholm D. (2020). Dynamic Interaction of USP14 with the Chaperone HSC70 Mediates Crosstalk between the Proteasome, ER Signaling, and Autophagy. iScience.

[62] Pantazopoulou M., Brembati V., Kanellidi A., Bousset L., Melki R., Stefanis L. (2021). Distinct alpha-Synuclein species induced by seeding are selectively cleared by the Lysosome or the Proteasome in neuronally differentiated SH-SY5Y cells. J. Neurochem..

[63] Sengupta S., Peterson T.R., Sabatini D.M. (2010). Regulation of the mTOR Complex 1 Pathway by Nutrients, Growth Factors, and Stress. Mol. Cell.

[64] Kim J., Kundu M., Viollet B., Guan K.-L. (2011). AMPK and mTOR regulate autophagy through direct phosphorylation of Ulk. Nat. Cell Biol..

[65] Alers S., Löffler A.S., Wesselborg S., Stork B. (2012). Role of AMPK-mTOR-Ulk1/2 in the regulation of autophagy: Cross talk, shortcuts, and feedbacks. Mol. Cell. Biol..

[66] Renna M. (2010). Chemical inducers of autophagy that enhance the clearance of mutant proteins in neurodegenerative dis-eases. J. Biol. Chem..

[67] Williams A. (2008). Novel targets for Huntington’s disease in an mTOR-independent autophagy pathway. Nat. Chem. Biol..

[68] Criollo A., Maiuri M.C., Tasdemir E., Vitale I., A Fiebig A., Andrews D., Molgó J., Díaz J., Lavandero S., Harper F. (2007). Regulation of autophagy by the inositol trisphosphate receptor. Cell Death Differ..

[69] Sarkar S., Floto R.A., Berger Z., Imarisio S., Cordenier A., Pasco M., Cook L.J., Rubinsztein D.C. (2005). Lithium induces autophagy by inhibiting inositol monophosphatase. J. Cell Biol..

[70] Motoi Y., Shimada K., Ishiguro K., Hattori N. (2014). Lithium and Autophagy. ACS Chem. Neurosci..

[71] Thellung S., Corsaro A., Nizzari M., Barbieri F., Florio T. (2019). Autophagy Activator Drugs: A New Opportunity in Neuroprotection from Misfolded Protein Toxicity. Int. J. Mol. Sci..

[72] Vakifahmetoglu-Norberg H., Xia H.-G., Yuan J. (2015). Pharmacologic agents targeting autophagy. J. Clin. Investig..

[73] Sharif N.A. (2018). iDrugs and iDevices Discovery Research: Preclinical Assays, Techniques, and Animal Model Studies for Ocular Hypotensives and Neuroprotectants. J. Ocul. Pharmacol. Ther..

[74] Sharif N.A. (2018). Glaucomatous optic neuropathy treatment options: The promise of novel therapeutics, techniques and tools to help preserve vision. Neural Regen. Res..

[75] Fuwa M., Kageyama M., Ohashi K., Sasaoka M., Sato R., Tanaka M., Tashiro K. (2019). Nafamostat and sepimostat identified as novel neuroprotective agents via NR2B N-methyl-D-aspartate receptor antagonism using a rat retinal excitotoxicity model. Sci. Rep..

[76] Sasaoka M., Ota T., Kageyama M. (2020). Rotenone-induced inner retinal degeneration via presynaptic activation of voltage-dependent sodium and L-type calcium channels in rats. Sci. Rep..

